# Filling Teeth

**Published:** 1866-08

**Authors:** J. Taft


DENTAL REGISTER.

VOL. XX.] AUGUST, 1866. [No. 8.

### Original Communications.

### FILLING TEETH.

BY J. TAFT.

The conditions that modify success in the operation of filling are not usually apparent to the uninitiated. The query is often put forth by the patient: "Why is it that teeth decay so often after they are filled—and even when well filled, too?" This question cannot always be answered to the entire comprehension of the querist; for very few indeed of those who have not made the subject a special study possess the knowledge that must antidate the full understanding of it. And again, he who attempts to answer may fail to make any point well understood, either from a want of knowledge or efficient descriptive ability.

Of the circumstances that modify the permanency of fillings none is more apparent than the manner of the performance.

Fillings should always be so introduced, that all foreign subtances will be effectually excluded from the cavity of decay, and so far as possible cover and protect every point of dentine exposed by decay. This perfect adaptation is not in a great many cases obtained without difficulty, even by the best skill and experience. As evidence of this, discoloration and decay frequently occur beneath what was supposed to be good fillings. It is usually a good indication to find the dentine retaining its color about a filling. Many operators give great care and attention to secure great density of their fillings, and still fail in obtaining a perfect adaptation of the filling to the walls of the cavity. There is not, as a general rule, proper care exercised in introducing the filling, some avowedly are not scrupulous in filling up the cavity, but claim by making a thorough adaptation of the gold to its border, that all other substances will be excluded and the case is safe.

This many a time proves false however, for it only requires a slight disturbance of the gold at the border of the filling, or a slight fracture of the edge of the dentine about it to insure the recurrence of decay, and the ultimate loss of the tooth, so far as that operation is concerned. While, on the other hand, if the adaptation has been perfect throughout the introduction of the gold, it may be roughened or broken up on the surface, or the edges of the cavity broken or worn down, and still nothing can pass into the cavity, and decay does not occur within it.

How important then that every operator should train himself in this particular. The manner of doing this will depend somewhat upon the material employed. In the use of adhesive gold perhaps a little more care is required to secure a perfect adaptation than with good non-adhesive, yet it can be accomplished with either by proper skill. In the introduction of adhesive gold, the ordinary round or square serrated instrument employed for condensing the body of the filling should not be relied upon for condensing to the walls of the cavity, but thin wedge shaped serrated instruments should be used; these should vary in width corresponding somewhat to the size of the cavity, forcing the gold most perfectly upon it, and especially upon approaching the orifice.

